# Structural and Functional Stability of DNA Nanopores in Biological Media

**DOI:** 10.3390/nano9040490

**Published:** 2019-03-29

**Authors:** Jonathan R. Burns, Stefan Howorka

**Affiliations:** 1 Department of Chemistry, Institute of Structural Molecular Biology, University College London, London WC1H 0AJ, UK; 2 Institute of Biophysics, Johannes Kepler University, A-4020 Linz, Austria

**Keywords:** DNA nanotechnology, nanopores, biological media, serum, stability, aggregation

## Abstract

DNA nanopores offer a unique nano-scale foothold at the membrane interface that can help advance the life sciences as biophysical research tools or gate-keepers for drug delivery. Biological applications require sufficient physiological stability and membrane activity for viable biological action. In this report, we determine essential parameters for efficient nanopore folding and membrane binding in biocompatible cell media. The parameters are identified for an archetypal DNA nanopore composed of six interwoven strands carrying cholesterol lipid anchors. Using gel electrophoresis and fluorescence spectroscopy, the nanostructures are found to assemble efficiently in cell media, such as LB and DMEM, and remain structurally stable at physiological temperatures. Furthermore, the pores’ oligomerization state is monitored using fluorescence spectroscopy and confocal microscopy. The pores remain predominately water-soluble over 24 h in all buffer systems, and were able to bind to lipid vesicles after 24 h to confirm membrane activity. However, the addition of fetal bovine serum to DMEM causes a significant reduction in nanopore activity. Serum proteins complex rapidly to the pore, most likely via ionic interactions, to reduce the effective nanopore concentration in solution. Our findings outline crucial conditions for maintaining lipidated DNA nanodevices, structurally and functionally intact in cell media, and pave the way for biological studies in the future.

## 1. Introduction

DNA nanotechnology excels at the bottom-up fabrication of engineered nanostructures. DNA duplexes can be manipulated into user-defined shapes by exploiting the base-pairing rules for duplex formation [1,2,3]. Discrete nanostructures can be assembled in two and three dimensions with sub-nanometer control using dedicated design software [4,5]. Chemical diversity and functionality can be incorporated into structures site-specifically using, for example, solid phase DNA synthesis [6,7], or non-specifically via intercalation [8,9], or electrostatic interactions [10,11]. This rapidly evolving field has transformed materials science with wide-ranging applications, including the generation of DNA origami devices for optical sensing [12], controlled single molecule synthesis using a lab-on-a-chip DNA board [13], computation devices [14,15], and finite sub-nm movement of DNA-based robots using DNA ligands [16]. 

DNA nanotechnology applied to the life sciences is gaining traction. DNA nanostructures can help control processes within cells, or at the membrane interface to advance biological understanding [17,18,19,20]. This progress includes the generation of novel diagnostic tools [21,22,23], the enhancement of existing drugs [24], and devices with novel therapeutic action [25]. Recently, intracellular DNA-based delivery vehicles have been used to transport biomolecules. Engineered DNA cages that encapsulate small molecule drugs [25], mRNA [26], peptides, and proteins [27,28] have been developed to deliver biomolecular cargo. DNA nanostructures can be internalized in specific mammalian cells, although the nature of the design appears to play an important role [29,30]. Coating the nanostructures in certain chemical groups can improve cellular uptake [10,31,32]. 

To fulfill desirable biomolecular functions, the DNA nanostructures have to be stable. DNA origami-based nanostructures have been studied previously in vitro and in vivo [33]. Generally, the origami constructs withstand diverse biology conditions under short time durations. Yan and colleagues have recently shown intact and functional DNA origami in the renal system of a mouse model [34]. However, other reports have identified significant degradation and unfolding of DNA origami structures in biological media [35,36]. This instability has been attributed to the low level of Mg^2+^ ions—essential to stabilize DNA origami nanostructures—and digestion from enzymes including DNAses. The susceptibility to degradation appears to be design-specific, with tubular designs proving more resilient [37]. Other strategies can be employed to help stabilize the nanostructures, including chemical ligation of DNA nicks [38], the introduction of non-native base pairs, such as LNA, PNA, and XNA [39]. Alternatively, cationic peptides [10], polymers [32], or intercalators [9] can be used to improve structural stability.

DNA-based nanopores are the most recent class of membrane channels which can potentially offer a unique degree of control at the membrane interface [40,41,42,43,44]. Naturally occurring nanopores are usually composed of proteins or peptides to help regulate ion transport across cell membranes [45]. However, it can be challenging to de novo design amino acid-based nanopores due to unexpected protein misfolding [46]. In contrast, utilizing DNA as a construction material can help overcome this issue. To date, DNA nanotechnology has produced nanopores with highly customizable properties, including channel diameter, length, functionalized groups within the lumen, and ligand-controlled pore opening [40,41,42,43,44]. For future biological applications, including pore-mediated drug delivery, nanopore stability and solubility within biological media must be maintained.

**Figure 1:**
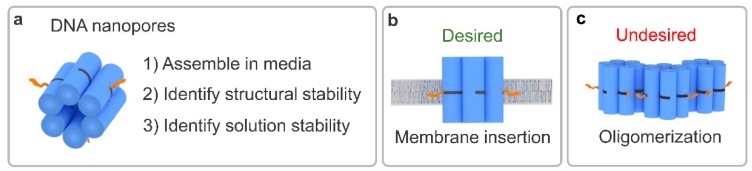
Identifying the formation, structural and solution-phase stability of amphiphilic DNA nanopores in biological media. (**a**) Depiction of six helical bundle nanopore (**blue cylinders**) containing cholesterol lipid anchors (**orange**) and the parameters monitored within; (**b**) the desired monomer membrane binding action in vivo; (**c**) and the undesired hydrophobic lipid anchor-mediated oligomerization which can prevent membrane binding and reduce the active nanopore concentration.

To investigate DNA nanopore stability, this study employs the DNA nanopore NP-3C (Figure 1a) [42]. The pore is assembled from six single strands (Tables S1 and S2, Figures S1 and S2), which form six interwoven DNA duplexes to generate a six helical barrel. Three cholesterol lipid anchors are site-specifically incorporated to the exterior of the bundle to facilitate membrane binding and nanopore behavior. The assembled pore punctures the membrane to generate a toroidal pore to enable ion transport across the lipid bilayer (Figure 1b) [44,47,48]. However, the hydrophobic lipid anchors can also mediate other undesired behavior, including intermolecular oligomerization (Figure 1c) [49,50]. To help distinguish the lipid anchor effect, a cholesterol-free version, NP-0C, was assembled to serve as a negative control (Tables S1 and S2, Figure S1).

Cell media is composed of complex ions and nutrients which help to maintain cell homeostasis and phenotype. For biological applications, the amphiphilic DNA nanostructures must remain structurally stable within the used medium. Therefore, this report assayed biologically compatible media to identify the pore’s structural stability and membrane activity, including phosphate-buffered saline (PBS), bacterial growth medium lysogeny broth (LB), mammalian cell media Dulbecco’s modified Eagle medium (DMEM), and DMEM supplemented with 10% *v*/*v* fetal bovine serum (FBS) (Table 1) [51,52]. Serum is required for specific cell types to maintain cell function, and is composed of a wide array of entities including proteins, hormones, and electrolytes. The total protein concentration in FBS is ~0.3–0.5 mg/mL [53]. Albumin, globulin, and fibrinogen make up the majority of proteins found in serum, at approximately 55, 38, and 7%, respectively. In addition, over a thousand other regulatory proteins exist at much smaller levels. Metal ions must also be considered. Positively charged metal ions coordinate with DNA ionically to stabilize duplexes. Therefore, a range of metal cations was assayed to identify the counterion stabilization on the nanostructures [33]. We tested monovalent sodium and potassium ions typically used for single channel current recordings used to study nanopores [54], and divalent magnesium ions, conventionally used for the stabilization of DNA origami constructs. The nanopore formation was determined using gel electrophoresis. To identify the thermal stability at physiological temperatures in biological media, the melting temperatures of the constructs were identified using fluorescence spectroscopy [55]. Further, our study identified the aggregation extent of the nanostructures using fluorescence spectroscopy and confocal microscopy over time. Finally, to confirm membrane activity of the nanopore, binding to model membranes was determined using fluorescence microscopy. With the knowledge gained using our approach, new pore formulations and folding protocols can be established which should help provide insights for future applications across the life sciences.

## 2. Materials and Methods

All reagents were purchased from Sigma Aldrich (UK) unless stated otherwise. The DNA nanopore was published previously (information on the sequences, including 2D maps and dimensions is provided in the Supporting Information) [42]. The DNA nanopores were assembled by mixing an equimolar mixture of the component DNA strands (0.5 µM, unless stated otherwise) (Integrated DNA Technologies, Coralville, IA, USA) containing the stated buffer or media. The nanopores were folded by heating the solution from 95 °C for 2 min, and cooling to 20 °C at a rate of 5 °C per min. The folded DNA nanopore constructs were stored at room temperature, and vortexed for 2 s before use. Where stated, *n*-octyl-oligo-oxyethylene (OPOE) (Enzo Life Sciences, Exeter, UK) was added to the folding mixture prior to nanopore assembly (1.5% *v*/*v*).

Buffer and reagents. Na: NaCl 300 mmol/L, tris 15 mmol/L, pH 8.0. K: KCl 300 mmol/L, tris 15 mmol/L, pH 8.0. Mg: MgCl_2_ 14 mmol/L, tris 40 mmol/L, acetic acid 20 mmol/L, ethylenediaminetetraacetic 1 mmol/L, pH 8.3. PBS: NaCl 137 mmol/L, KCl 2.7 mmol/L, Na_2_HPO_4_ 8 mmol/L, KH_2_PO_4_ 2 mmol/L, pH 7.4. LB: tryptone 10 mg/mL; yeast extract 5 mg/mL; NaCl 10 mg/mL values taken from [56]. D components include CaCl_2_ 2.4 mmol/L, MgSO_4_ 0.8 mmol/L, KCl 5.4 mmol/L, NaHCO_3_ 44.0 mmol/L, NaCl 109.5 mmol/L, NaH_2_PO_4_ 0.9 mmol/L. Neat fetal bovine serum components include bilirubin 2.4 mg/L; Cholesterol 340 mg/L; creatinine 27.3 mg/L; urea 260 mg/L; Na^+^ 142 mmol/L; Cl^−^ 155.5 mmol/L; K^+^ 8 mmol/L; Ca^2+^ 3 mmol/L; Mg^2+^ 1.1 mmol/L; PO_4_^3−^ 2.3 mmol/L; Fe 1.6 mg/L; glucose 550 mg/L; protein 36 g/L; albumin 17 g/L; α-globulin 17 g/L; β-globulin 2 g/L; γ-globulin 1 g/L, values taken from [57].

DNA nanopore folding was characterized using 12% sodium dodecyl sulfate polyacrylamide gel electrophoresis (SDS-PAGE) (Bio-Rad, Watford, UK) with standardized buffers typically applied to proteins. The gel was thermally equilibrated at 8 °C prior to loading. The gel was run at 140 V for 70 min. The bands were visualized by first removing SDS with deionized water, then stained using ethidium bromide solution. A 100 base-pair DNA marker (New England Biolabs, Hitchin, UK) was used as a reference.

The Förster resonance energy transfer (FRET) characteristics of the fluorescein (FAM) and Cy3 labeled nanopore constructs were identified using a Varian Eclipse fluorescence spectrophotometer (Agilent, Stockport, UK). 20 µL of the various DNA nanostructures (folded in PBS at 1 µM) (see Tables S1 and S2 for strand information) was added to PBS (180 µL) in a quartz cuvette with a path length of 1 cm. The samples were analyzed by excitation at 495 nm, and the emission monitored between 505–700 nm. A 5 nm slit width and 600 PMT voltage was applied, along with a scanning rate of 600 nm per min; the scan was performed 3 times and averaged.

The melting transitions of the DNA nanostructures were identified using a MyIQ real-time PCR (Bio-Rad, Watford, UK). The nanostructures were assembled containing FAM and Cy3 FRET pairs (folded at 1 µM in PBS). The DNA constructs were diluted into the stated buffer systems to give a final DNA concentration of 0.1 µM (total volume of 25 µL) in a 96-well thin wall fluorescence plate (Bio-Rad, Watford, UK). Optical quality sealing tape (Bio-Rad, Watford, UK) was placed on top to prevent evaporation. The sample was heated from 30–85 °C at a rate of 0.5 °C per min. The melting temperature was determined from taking the derivative of the donor fluorescence profile. Errors were identified from 3 independent experiments.

Fluorescence spectroscopic analysis was performed on Cy3-modified DNA nanostructures using a Varian Eclipse fluorescence spectrophotometer (Agilent, Stockport, UK) with a fluorescence cuvette. The samples were analyzed by excitation at 540 nm, and by monitoring the emission from 550–600 nm, using a 10 nm slit width, 800 PMT voltage, scanning at 600 nm per min and taking the average of 3 repeat scans. The DNA nanostructures (2 µL, folded at 0.5 µM) in the stated buffers were scanned once the folding temperature reached 40 °C by diluting in the buffer systems (200 µL final volume). At the designated time points, the samples were centrifuged for 10 min at 16k revolutions per min at room temperature (Eppendorf, Stevenage, UK), and the supernatant was carefully extracted and the fluorescence monitored using the same dilution and settings as described.

Confocal laser scanning microscopy (CLSM) images were collected using a 60× oil objective FV-1000 Olympus microscope. Images were analyzed using ImageJ software. To image the DNA nanopore constructs, the folded pore containing a Cy3 dye (10 µL, 0.5 µM in PBS) was deposited on a fluorodish (World Precision Instruments, Sarasota, FL, USA), and left to settle for 20 min prior to imaging. For the vesicle-binding assays, 1-palmitoyl-2-oleoyl-sn-glycero-3-phosphocholine (POPC) giant unilamellar vesicles (GUVs) were prepared by modifying a published protocol [48,58]. POPC (150 µL, 10 mM) in chloroform was added to a 1 mL glass vial, the solvent was removed under vacuum, and underwent rotation using a rotary evaporator. The thin film generated was resuspended in mineral oil (150 µL) by vortexing and sonicating for 10 min. Green fluorescent protein (5 µL,10 µM in PBS) was mixed with sucrose solution (20 µL, 400 mM), followed by addition of mineral oil (150 µL). The suspension was vortexed and sonicated for 10 min at room temperature, then carefully added to the top of a glucose solution (1 mL, 400 mM) in a plastic vial (1 mL). The vesicles were generated by centrifuging at 16K RPM at 4 °C for 10 min. The mineral oil top layer and the majority of the sucrose layer (~800 µL) were carefully removed. The remaining solution containing the pelleted vesicles was gently mixed with a pipettor, then transferred to a clean plastic vial. The POPC GUVs (5 µL) were added to a KCl solution (5 µL, 0.5 M), and then Cy3-labeled NP-3C (2 µL folded at 0.25 µM) in the stated buffers was mixed, and the solution deposited on the confocal slide and used within 24 h. For the serum time series assay, NP-3C (10 µL, 0.25 µM folded in PBS) was added to FBS (10 µL) for the stated time durations. The NP-3C-FBS solution (4 µL) was added to the GUV solution as described above. All images were collected after 20 min using identical settings.

## 3. Results

### 3.1. Determining Nanopore Formation in Media

**Figure 2:**
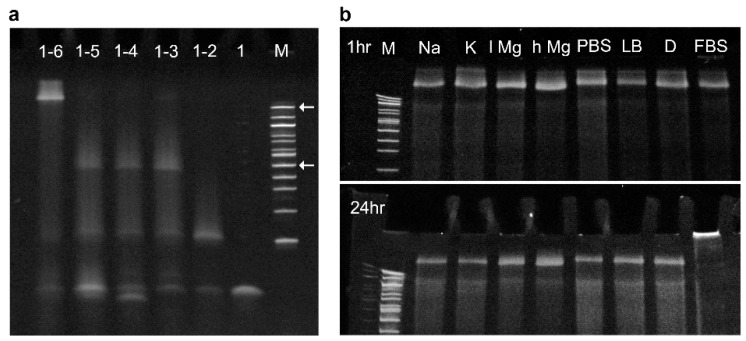
The DNA nanopores fold efficiently and remain structurally stable in a range of salts and buffer conditions as shown by gel electrophoresis. Sodium dodecyl sulfate polyacrylamide gel electrophoresis (SDS-PAGE) reveals (**a**) step-wise assembly of NP-0C, left to right, fully assembled barrel strands (**1–6**), followed by component strands (**1–5**), (**1–4**), (**1–3**), (**1–2**), (**1**), and 100 base pair marker (**M**), the arrows indicate the 1517 (**top**) and 500 base pair (**bottom**) marker bands; (**b**) 1 h (**top**) and 24 h (**bottom**) after folding of NP-0C assembled in a variety of conditions; 100 base pair marker (**M**), 0.3 M sodium chloride (**Na**), 0.3 M potassium chloride (**K**), 14 mM magnesium chloride (**l Mg**), 140 mM magnesium chloride (**h Mg**), phosphate buffered solution (**PBS**), Lysogeny Broth (**LB**), Dulbecco’s modified Eagle medium (**D**), and Dulbecco’s modified Eagle medium supplemented with 10% fetal bovine serum (**FBS**).

The folding efficiency of the DNA nanopores in biological media was analyzed using SDS-PAGE (Figure 2). First, the formation of the folded barrel was confirmed by assembling NP-0C, as well as versions missing some of the component strands (Figure 2a). Combining all six strands yielded the slowest migrating band slightly above the 1517 base pair marker band, as indicated by the top arrow. Removal of a single strand from the folding mixture resulted in an increase in band mobility. The 5-component construct migrated towards the 500 base pair marker band, as indicated by the bottom arrow. The large shift in band migration between a fully and partially assembled barrel is consistent with the formation of a higher order tertiary nanostructure. However, the strand combinations 1–4 and 1–3 also gave rise to a band migrating aligned to the 500 bp marker band. This result indicates that the addition of strands 4 and 5 to the pooled mixture did not successfully incorporate within the assembled bundle. Comparing component strands 1–2 and 1 gave the expected step-wise change indicating successful assembly.

The assembled NP-0C construct folded efficiently in all biological buffers as assayed by SDS-PAGE (Figure 2b). This result confirmed stable DNA nanostructure formation in diverse media over short time durations, even in the presence of low salt conditions. After 24 h the gel was repeated, all bands showed very similar behavior—except for the media containing FBS—indicating that the pores are generally stable under these varied conditions. Adding protein-containing FBS led to the formation of protein-DNA complexes that did not migrate into the gel. The surfactant in the gel buffer, sodium dodecyl sulfate (SDS), was not able to disrupt the protein binding to the DNA nanostructure.

### 3.2. Identifying Nanopore Melting Temperatures in Biological Media

**Figure 3:**
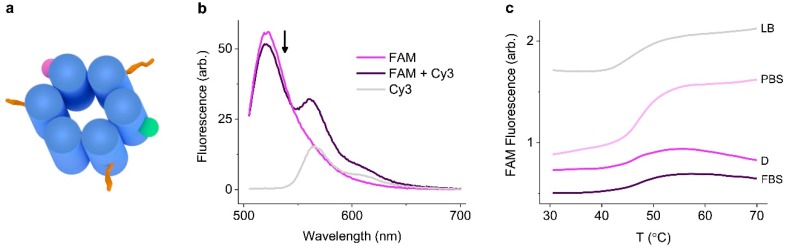
Determining the thermal stability of DNA nanopores in biological media using the Förster resonance energy transfer (FRET) pair labeled DNA nanopores. (**a**) Representation of fluorescein (FAM) (**purple**) and Cy3 (**green**) fluorophores incorporated into the DNA nanopore constructs; (**b**) fluorescence emission spectra of FAM (donor) and Cy3 (acceptor) labeled DNA nanopores, excitation at 495 nm, the donor emission is decreased in the presence of the acceptor in the assemble pore construct; (**c**) fluorescence donor emission thermal melting profiles of NP-3C in the stated buffers. The different fluorescence intensities reflect how the applied buffer system influence fluorophore emission.

The thermal stability of the pores was established using DNA nanostructures labeled with a fluorophore pair for Förster resonance energy transfer (FRET). FAM (fluorescein) (donor) and Cy3 (acceptor) FRET pairs [59] were incorporated into the nanostructures on strands 2 and 6, respectively (see the Supporting Information for details for the DNA strands) (Figure 3a). Successful FRET was confirmed by scanning the donor emission in the absence and presence of the acceptor in assembled NP-3C (Figure 3b). Next, the donor emission was monitored upon heating (Figure 3c). The donor profile gave rise to a sigmoidal curve and its derivative yielded the melting transition. All constructs in the media types displayed melting transitions significantly above physiological temperatures (Table 2). Monovalent sodium and potassium gave very similar melting transitions for NP-3C, at 51.3 °C and 52.2 °C, respectively. However, divalent magnesium gave rise to a 1.6 °C enhancement, even though the counterion concentration was significantly lower. Biologically compatible PBS reduced the structural stability by 4 °C, possibly due to the lower concentration of monovalent sodium (137 mM). However, the overall high thermal stability of all the nanostructures in all the media conditions confirmed their suitability for biological applications from a structural perspective.

### 3.3. Identifying Time-Dependent Nanopore Water-Solubility

**Figure 4:**
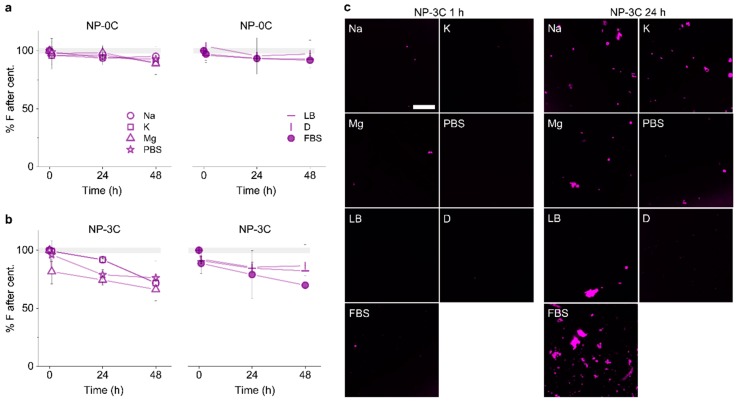
Identifying aggregation of DNA nanopores over time. Relative fluorescence intensities of Cy3-labeled (**a**) NP-0C and (**b**) NP-3C remaining in solution after centrifugation at 1, 24, and 48 h, in the stated buffers; (**c**) CLSM images of Cy3-labeled NP-3C in the stated buffers at 1 and 24 h. Scale bar 50 µm.

We tested the water solubility of constructs in the different media conditions. Centrifugation was used to pellet and separate any large NP-3C clusters from smaller water-soluble fractions. The fluorescence in the supernatant was quantified using a fluorometer (Figure 4a), and fluorescence in the pellet using confocal laser scanning microscopy (CLSM) (Figure 4b). The cholesterol-free construct remained predominantly water-soluble over the course of 48 h in all assayed media. The cholesterol labeled nanopore, NP-3C, showed some aggregation and pelleting after 24 h. However, the majority of the oligomerized and monomeric form remained water soluble (>75%) even after 48 h. The protein-DNA complexes generated by FBS resulted in a noticeable increase in the pelleting fraction; however, the majority of complexed NP-3C in FBS remained water-soluble. By comparison, NP-0C showed no aggregation either in the supernatant, or CLSM images after 48 h across all conditions. This result confirms the cholesterol lipid anchors were responsible for the detectable pelleting observed.

### 3.4. Identifying Nanopore Membrane Binding Activity in Media

**Figure 5:**
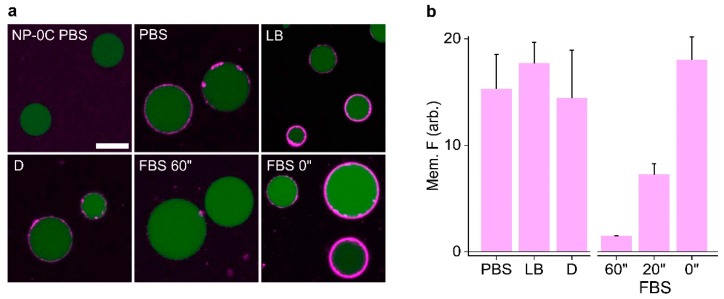
Confocal microscopy shows NP-3C binding to GUVs in biological buffers while FBS complexes to NP-3C to block membrane tethering. (**a**) Cy3-labeled NP-3C (**magenta**) mixed in the stated buffers for 1 h, and NP-3C added to FBS for different time periods, then added to GUVs containing GFP (**green**), image collected after 20mins of pore-vesicle incubation on a glass slide; (**b**) relative membrane fluorescence intensities from (**a**). All images collected under identical conditions. Error bars represent the averages of three experiments. Scale bar 10 µm.

We tested the ability of DNA nanopores to bind to vesicles in media. The DNA nanopores binding towards giant unilamellar vesicles (GUVs) was identified using CLSM (Figure 5). Green fluorescence protein (GFP) was encapsulated inside the GUVs to aid visualization. Cy3-labeled NP-3C in PBS, LB, or DMEM was diluted into the GUV solution to minimize the effect different salts and buffers have on dye fluorescence. Extensive membrane binding to GUVs was observed for these combinations, as shown by intense membrane halos around the vesicles’ perimeter. In contrast, NP-3C in serum-containing media blocked the nanopores’ binding event significantly. In agreement with SDS-PAGE analysis described above, FBS proteins complexed to the pore, via the generation of a higher molecular weight complex which prevents cholesterol-mediated binding. However, this problem can be circumvented by minimizing the pores exposure to FBS prior to vesicle addition. Adding the pores to FBS for 20 min, or directly from PBS, followed by addition to GUVs resulted in significant membrane binding, comparable to the other media conditions. This is an important finding for future lipidated DNA nanodevices employed in serum-containing media.

### 3.5. Adding Detergent Prevents Nanopore Aggregation

**Figure 6:**
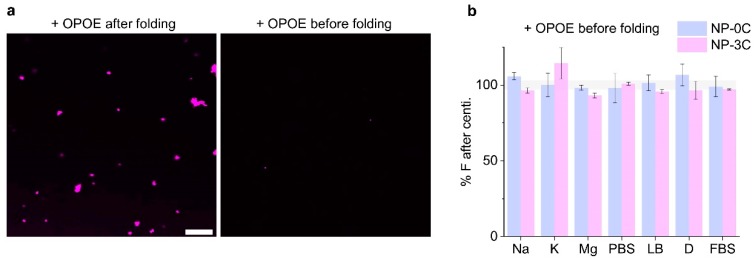
Adding surfactant prevents nanopore aggregation and precipitation. (**a**) CLSM images of Cy3-labeled NP-3C (**magenta**) in FBS 48 h after folding, with OPOE added at 48 h (**left**), or prior to folding (**right**), all images collected under identical conditions, scale bar 50 µm; (**b**) fluorescence analysis of the supernatant fraction after centrifugation of NP-0C (**blue**) and NP-3C (**magenta**) folded in the presence of OPOE. Error bars represent the averages of three independent experiments.

We employed the non-ionic surfactant *n*-octyl-oligo-oxyethylene (OPOE) to generate a DNA nanopore-detergent complex to improve solubility and prevent aggregation. Naturally occurring membrane proteins are amphiphilic and can aggregate due to poor aqueous solubility. To help overcome this issue surfactants are routinely utilized to extract, solubilize, and stabilize membrane proteins [60]. OPOE was previously shown to aid DNA nanopore insertion into membranes during single channel current recordings [42]. We tested whether the surfactant can reverse pre-aggregation of nanopores using the centrifugation assay described above. The surfactant was added in high concentrations above the critical micelle concentration (CMC). OPOE was added after incubating the pore 48 h in FBS. CLSM revealed NP-3C aggregates after the addition of OPOE (Figure 6a) suggesting that the surfactant was not able to disrupt the pre-formed NP-3C aggregate. However, when the detergent was added to the pooled DNA prior to assembly, no aggregates were observed either by confocal microscopy, or a decrease in the supernatant fluorescence after 48 h (Figure 6a,b). These results confirm that the mild surfactant was able to solubilize the pores and prevent higher-order assembly for long durations. These results indicate that future folding protocols should include detergent within the folding mixture, prior to, or shortly after nanopore folding to help prevent lipidated DNA nanostructure aggregation.

## 4. Discussion

DNA-based nanopores are a recent and exciting class of synthetic membrane channel. This construction approach provides a unique level of biophysical control across lipid bilayers. For DNA nanopores to provide functionality in the life sciences, for example, as biosensors, drug delivery vehicles, or cytotoxic-inducing agents, biocompatibility needs to be addressed. Essential parameters before in vitro and in vivo testing include confirming structural stability and solution-phase solubility in biologically compatible media. We have employed an archetypical DNA nanopore and tested its folding capabilities in diverse biological media routinely used to culture mammalian or bacterial cells. The conditions were deliberately chosen to be stringent as folding was performed in the cell media. It would also have been possible to fold pores in protein-free buffers and then add them to the protein-containing media. This differential treatment would have, however, made a fair comparison across all buffers and media more difficult. Our results suggest that pores folded efficiently and remained structurally stable in all media assayed. Importantly, the pore constructs displayed melting temperatures above physiological temperatures, even in the absence of divalent magnesium ions. With the exception of FBS-containing media, the DNA nanopores remained predominately water soluble in all conditions tested over 48 h. These results reinforce the suitability of DNA nanotechnology as a good building material for use in biological studies.

Serum proteins cause aggregation most likely due to complexation towards the DNA’s negatively charged phosphate backbone. Albumin serum proteins have been shown to complex to antisense oligonucleotides, which in some instances enhances the half-life of intravenously injected DNA [61,62]. However, hydrophobic groups can further increase the protein complexation extent [63]. In the case of amphiphilic DNA nanopores used in this study, the resultant complexes caused significant aggregation, drastically reducing the amount of membrane tethering action. This result is an important finding in the field of lipidated DNA nanostructures, and if the use of FBS is unavoidable, the DNA nanopores should be applied for short time periods of less than 1 h, or the cells transferred temporarily into other media, such as phosphate buffered saline. Alternatively, vesicle delivery agents may be employed to shield the nanostructures from aggregation. Other strategies include using DNA masking groups, such as coating the structures in polyethylene glycol, or carboxylic acids, both are known to improve the circulation time of biomolecules in the bloodstream. Cationic groups such as lysine and arginine-rich peptides [32], polyamines [64], and metal cations can be used to block the serum proteins from binding. In addition, charge-neutral peptide nucleic acids nanopore equivalents [39] can be developed to prevent serum complexation. We expect lipidated DNA nanodevices and DNA nanopores to provide a useful foothold cell biology, and should find use as drug delivery gate-keepers, to function as drug molecules, such as immunosuppressants or immunoactivators, or act as novel tools in diagnostics and sensing.

## Figures and Tables

**Table 1:** Buffer solutions and biological media including their ionic strength.

Abbreviation	Na	K	Mg	PBS	LB	D	FBS
Salt/media	NaCl	KCl	MgCl_2_ TAE	Phosphate buffered-saline	Lysogeny broth	Dulbecco’s modified Eagle medium	D + 10% fetal bovine serum
Ionic strength	0.32 M	0.32 M	0.11 M	0.17 M	0.17 M	0.17 M	0.19 M

**Table 2:** Melting temperatures of DNA nanopore constructs in stated salt and media systems. Errors were identified from three independent experiments.

Construct	Na	K	Mg	PBS	LB	D	FBS
NP-0C	49.7 ± 0.3	50.9 ± 1.2	52.7 ± 0.3	46.4 ± 0.9	46.6 ± 0.3	45.8 ± 0.3	45.7 ± 0.3
NP-3C	51.3 ± 0.6	52.2 ± 0.8	53.8 ± 1.5	46.7 ± 0.3	45.0 ± 1.0	46.8 ± 0.3	47.2 ± 0.3

## Supplementary Materials

The following are available online at https://www.mdpi.com/2079-4991/9/4/490/s1, Figure S1: 2D maps and Figure S2: graphical representation of constructs, Table S1: DNA sequences and Table S2: DNA strand combinations.
