# Supplementary material for: Structural and Functional Stability of DNA Nanopores in Biological Media
Source: Nanomaterials (Basel). 2019 Mar 29;9(4):490. doi: 10.3390/nano9040490 (PMC6523550; doi:10.3390/nano9040490)
Supplement: Supplementary file 1 [file nanomaterials-09-00490-s001.pdf]

Supporting Information

Structural and Functional Stability of DNA Nanopores in Biological Media

Jonathan R. Burns [1,*] and Stefan Howorka [1,2,*]

[1] Department of Chemistry, Institute of Structural Molecular Biology, University College London, London WC1H 0AJ, UK
[2] Institute of Biophysics, Johannes Kepler University, A-4020 Linz, Austria
* Correspondence: jonathan.burns@ucl.ac.uk (J.R.B.); s.howorka@ucl.ac.uk (S.H.)

Contents

1. Design of DNA nanopores 3

1.1. Sequences 3

1.2. 2D maps of DNA nanopores 3

1.3. Models and dimensions of DNA nanopores 4

1. Design of DNA nanopores

1.1. Sequences

Table S1. Names, chemical modifications, and sequences of DNA oligonucleotides used to prepare DNA nanopores.

ID	Sequence 5′ → 3′
1	AGCGAACGTGGATTTTGTCCGACATCGGCAAGCTCCCTTTTTCGACTATT
2	CCGATGTCGGACTTTTACACGATCTTCGCCTGCTGGGTTTTGGGAGCTTG
3	CGAAGATCGTGTTTTTCCACAGTTGATTGCCCTTCACTTTTCCCAGCAGG
4	AATCAACTGTGGTTTTTCTCACTGGTGATTAGAATGCTTTTGTGAAGGGC
5	TCACCAGTGAGATTTTTGTCGTACCAGGTGCATGGATTTTTGCATTCTAA
6	CCTGGTACGACATTTTTCCACGTTCGCTAATAGTCGATTTTATCCATGCA
1(chol)	Sequence of 1, carries a cholesterol via a tri(ethylene glycol) linker at the 3′ end
3(chol)	Sequence of 3, carries a cholesterol via a TEG linker at the 3′ terminus
5(chol)	Sequence of 5 carries a cholesterol via a TEG linker at the 3′ terminus

For FRET assays, strand 2 contained a FAM dye, whilst strand 6 contained a Cy3 dye. Both dyes were incorporated into the 5' terminus of the respective oligonucleotides.

Table S2. Names and composition of DNA nanopores and control nanostructures.

Nanopore	Oligonucleotides used
NP-0C	1, 2, 3, 4, 5, 6
NP-3C	1(chol), 2, 3(chol), 4, 5(chol), 6

1.2. 2D Maps of DNA Nanopores

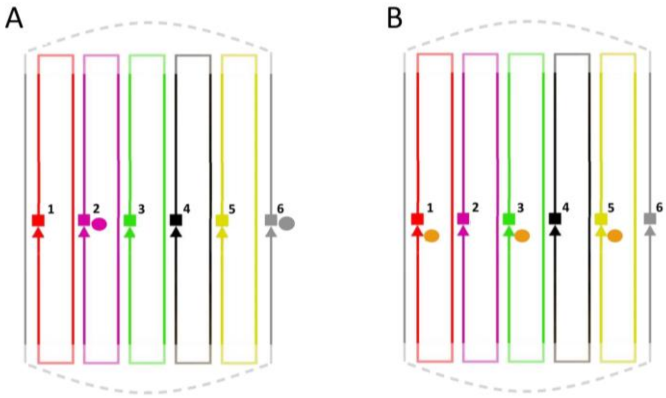

Figure S1. 2D maps of DNA nanopores (**A**) NP-0C and (**B**) NP-3C. The component DNA strands are represented as lines, and the 5' and 3' termini of the strands are indicated by squares and triangles, respectively. The segments in semi-transparent color at the top and bottom of the 2D maps indicate the mismatched T_4 single-strand loops. Orange circles show the positions for the cholesterol modifications, the purple and gray circles denote the position of the fluorophores.

1.3. Models and Dimensions of DNA Nanopores

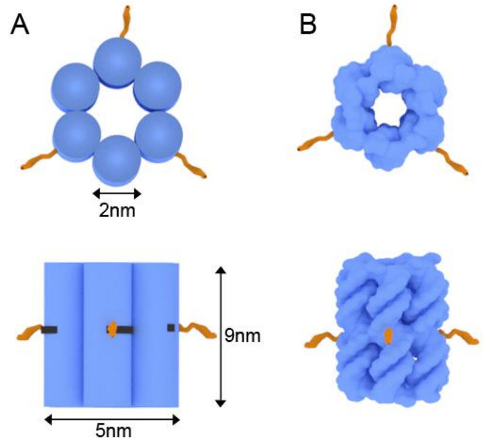

Figure S2. (**A**) Cylinder representation, top and side view of NP-3C (**B**) Space filling model representation, top and side view of NP-3C.
